# Association of intestinal barrier impairment with symptom severity and washed microbiota transplantation outcomes in atopic dermatitis patients

**DOI:** 10.3389/falgy.2025.1680658

**Published:** 2025-12-09

**Authors:** Ye-Dong Lao, Hong-Ying Zhang, Yao Cai, Min-Min Chen, Li-Hao Wu, Xing-Xiang He, Wan-Ying Deng

**Affiliations:** 1 Department of Gastroenterology, The First Affiliated Hospital of Guangdong Pharmaceutical University, Guangzhou, China; 2 Department of Gastroenterology, Research Center for Engineering Techniques of Microbiota-Targeted Therapies of Guangdong Province, The First Affiliated Hospital of Guangdong Pharmaceutical University, Guangzhou, China; 3 Department of Dermatology, The First Affiliated Hospital of Guangdong Pharmaceutical University, Guangzhou, China

**Keywords:** washed microbiota transplantation, WMT, intestinal barrier, atopic dermatitis, D-lactate

## Abstract

**Objective:**

We hypothesized that intestinal barrier impairment is a key pathophysiological feature in AD and that the degree of baseline barrier dysfunction, reflected by serum D-lactate levels, predicts the clinical response to Washed Microbiota Transplantation (WMT). This study aimed to test these hypotheses by investigating the association between intestinal barrier biomarkers and AD severity, and their correlation with WMT outcomes.

**Methods:**

We compared intestinal barrier biomarkers (D-lactate, endotoxin, and diamine oxidase) between 24 AD patients and 23 healthy donors. Additionally, we evaluated the clinical outcomes of 14 AD patients who underwent WMT therapy.

**Results:**

AD patients exhibited significantly elevated intestinal barrier biomarkers compared to healthy donors (*p* < 0.01). Following WMT, significant improvements were observed in SCORAD, EASI, and NRS scores (*p* < 0.05). In exploratory, uncorrected analyses, baseline D-lactate levels showed a significant negative correlation with improvements in SCORAD (*R* = −0.738, *p* = 0.037) and NRS scores (*R* = −0.650, *p* = 0.012), suggesting that higher pre-treatment levels might predict greater symptom relief. Microbiota analysis revealed a increase in Acidaminococcus and decreases in *Ruminococcus_gnavus_group*, *Flavonifractor,* and *Norank_f_Oscillospiraceae* following WMT.

**Conclusion:**

This study confirms significant intestinal barrier dysfunction in AD and demonstrates the potential clinical efficacy of WMT. The strong, uncorrected correlations suggest that pre-treatment D-lactate level warrants further investigation as a candidate biomarker for predicting WMT response. The clinical benefits occurred alongside a restructuring of the gut microbiota.

## 1 Introduction

Atopic dermatitis (AD) is a chronic, relapsing inflammatory skin disease characterized by intense pruritus, eczematous lesions, and a substantial impairment of quality of life (1). The pathogenesis of AD is complex, involving skin barrier dysfunction, immune dysregulation, and as increasingly recognized microbial dysbiosis (2, 3).

While historical research focused on the cutaneous microbiome, a growing body of evidence underscores the pivotal role of the gut microbiota and the bidirectional communication along the gut-skin axis in AD pathophysiology (4, 5). Patients with AD exhibit distinct gut microbial dysbiosis, including alterations in the abundance of genera such as *Bifidobacterium*, *Lactobacillus*, and *Faecalibacterium* (6, 7). This dysbiosis is believed to contribute to systemic inflammation and immune deviation, thereby influencing skin health (8). Supporting this concept, interventions targeting the gut microbiome, such as probiotics and prebiotics, have demonstrated efficacy in alleviating AD symptoms (9).

Fecal microbiota transplantation (FMT) represents a more comprehensive approach to restoring gut microbial homeostasis. Washed Microbiota Transplantation (WMT) is a novel concept and technique derived from FMT. Unlike traditional FMT processes, which rely on manual preparation of fecal samples, WMT automatically enriches microbiota from feces while incorporating a “washing” step. Studies have shown that this washing process removes certain bacterial fragments, pro-inflammatory metabolites, soluble molecules, and viruses, significantly reducing the probability of adverse events such as mucosal barrier damage and fever in patients (10, 11). Preliminary evidence, including work from our team, suggests that WMT can yield clinical benefits in AD patients (12). A critical component of the gut-skin axis is the intestinal barrier, which prevents the translocation of microbes and their products into systemic circulation. The integrity of this barrier can be assessed non-invasively by circulating biomarkers, including D-lactate, diamine oxidase (DAO), and endotoxin, which are elevated in states of increased intestinal permeability (13–15). Intestinal barrier dysfunction has been implicated in AD, yet its relationship with clinical severity and, more importantly, its role in predicting response to microbiota-targeted therapies remain poorly understood (16, 17).

Despite these advances, a significant translational gap persists. Currently, there are no validated biomarkers to predict which AD patients are most likely to respond to interventions like WMT, hindering the development of personalized treatment strategies. Furthermore, the specific impact of WMT on intestinal barrier function in AD has not been systematically evaluated.

Based on this evidence, we posited that intestinal barrier dysfunction is a prevalent and mechanistically important feature in AD patients. Furthermore, we proposed a novel therapeutic hypothesis: the very state of increased intestinal permeability, while detrimental in the chronic phase of AD, might paradoxically facilitate the initial efficacy of WMT by allowing beneficial microbial metabolites derived from the transplanted microbiota to more readily access systemic circulation and exert immunomodulatory effects. Therefore, we specifically hypothesized that: 1. AD patients would exhibit elevated serum levels of intestinal barrier biomarkers (D-lactate, endotoxin, DAO) compared to healthy donors. 2. Higher pre-treatment levels of these biomarkers, particularly D-lactate, would be associated with a greater clinical response to WMT.

## 2 Subjects and methods

### 2.1 Research objective

This study comprised two parts. The first part aimed to compare the biochemical markers of intestinal barrier function between AD patients and healthy donors. The second part was an interventional study to evaluate the efficacy of washed microbiota transplantation (WMT) in AD patients over a 4-week period. The pre-specified primary endpoint was the change in the SCORAD score. Secondary endpoints included changes in EASI, NRS, and DLQI scores; alterations in intestinal barrier biomarkers (D-lactate, endotoxin, diamine oxidase); and shifts in gut microbiota composition. Fecal samples were collected before (W0) and 4 weeks after (W1) WMT for microbiota analysis. The established Minimal Clinically Important Difference (MCID) thresholds were: SCORAD ≥8.7, EASI ≥6.6, NRS ≥4, and DLQI ≥4 (18–20).

### 2.2 Study participants

The study protocol was reviewed and approved by the Ethics Committee of The First Affiliated Hospital of Guangdong Pharmaceutical University [Approval No. [2023] IIT (12)]. Written informed consent was obtained from all participants or their legal guardians (for minor participants) prior to their enrollment in the study.

Study 1: From January 2021 to April 2024, a total of 48 participants under the age of 30 were included in the study: 24 AD patients and 23 fecal microbiota transplantation donors (healthy controls) recruited from the First Affiliated Hospital of Guangdong Pharmaceutical University between January 2021 and April 2024.

Study 2: 14 AD patients who underwent washed microbiota transplantation in the First Affiliated Hospital of Guangdong Pharmaceutical University during the same period were continuously included in the study.

Exclusion criteria: participants with severe gastrointestinal organic lesions such as Crohn's disease, gastrointestinal malignant tumors, no serious psychosomatic diseases such as malignant tumors, autoimmune diseases and other serious systemic organic lesions and mental diseases. Additionally, participants who had used antibiotics or probiotics within the 4-week period preceding the intestinal barrier testing or during the 4-week period surrounding WMT treatment (both pre- and post-procedure) were excluded.

### 2.3 Research methods

#### 2.3.1 Detection method of biochemical markers of intestinal barrier function

Measurement of intestinal barrier biochemical markers was performed within two days before and four weeks after microbiota transplantation therapy in AD patients. The Intestinal Barrier Function Biochemical Indicator Analysis System (model: JY-DLT) and the corresponding assay kits, manufactured by Beijing Zhongsheng Jinyu Diagnostic Technology Co., Ltd., were used for the detection. The Intestinal Barrier Function Biochemical Indicator Analysis System (model: JY-DLT) and corresponding assay kits, manufactured by Beijing Zhongsheng Jinyu Diagnostic Technology Co., Ltd., were used for the detection. According to the analytical performance data provided by the manufacturer, the assay parameters for each biomarker were as follows: diamine oxidase had a linear range of 5–80 U/L with a limit of blank (LoB) ≤4 U/L; D-lactate had a linear range of 2–50 mg/L with a LoB ≤1 mg/L; endotoxin had a linear range of 10–160 U/L with a LoB ≤8 U/L. The coefficient of variation (CV) for all assays was ≤15%. According to the manufacturer's instructions for the assay kits, the normal reference values were defined as: DAO ≤10 U/L, D-lactate ≤15 mg/L (corrected from U/L, assuming typo based on Table 1), and lipopolysaccharide (endotoxin) ≤20 U/L (21–23).

**Table 1:** Demographic characteristics.

Characteristic	AD patient (*n* = 24)	Donor (*n* = 23)	AD patients receiving WMT (*n* = 14)
Age (years)	21.16 ± 4.57	17.04 ± 7.96	26.85 ± 17.18
Gender (male)	19 (79.2%)	14 (60.8%)	10 (71.4%)
Nausea or/and vomiting	0	0	0
Decreased or absent bowel sounds	0	0	0
Diarrhea	0	0	0

The process was as follows: 1. 4 mL peripheral venous blood was collected after fasting for 8 h; 2. The blood samples were placed in a low temperature refrigerator at 4–6 °C, and the conventional standing time was not more than 4 h; 3. DAO, D-lactate and lipopolysaccharide were detected according to the standard process provided by the manufacturer. Patient baseline information and clinical symptoms and signs within 48 h prior to testing were recorded. The report form was automatically generated by the computer.

#### 2.3.2 Donor screening and washed microbiota transplantation (WMT) procedure

In this study, we defined the donor inclusion criteria for this study according to the Nanjing Consensus on Washed Microbiota Transplantation. All fecal microbiota donors were rigorously screened and selected in strict accordance with the Nanjing Consensus on Methodology of Washed Microbiota Transplantation through a multi-stage screening process to exclude individuals at risk of infectious diseases or microbiota-associated disorders. The screening process included initial exclusion of candidates with relevant medical histories and lifestyle risk factors through questionnaire assessment, followed by interview screening conducted by qualified physicians to further evaluate psychological status and potential risks, and comprehensive laboratory testing completed within 3 weeks prior to donation. The laboratory testing encompassed blood tests (screening for human immunodeficiency virus, hepatitis B and C viruses, Epstein–Barr virus, cytomegalovirus, and syphilis) and stool tests (including *Clostridioides, difficile*, *Salmonella*, *Shigella*, *Campylobacter* and other enteric pathogens). Additionally, a rapid questionnaire was administered on each donation day to exclude any temporary risk factors. Only donors who successfully passed all stages of this screening protocol were deemed eligible for donation.

The treatment procedure for washed microbiota transplantation comprised three main stages: preparation of the bacterial suspension, preparation of the intestinal catheter, and administration of the suspension. First, screened qualified donor feces were mixed with sterile saline in a 1:5 (feces:saline) volume ratio, thoroughly stirred, and allowed to stand for 5 min. The mixture was then filtered through a microporous filter membrane and aliquoted. Subsequently, the sub-packed samples were centrifuged and purified: the centrifuge was run at 2,500 rpm for 3 min at room temperature, and 30–40 mL of sterile saline was added to re-suspend after the clarification liquid layer was discarded. This centrifugation-washing process requires two cycles of operation to complete the sample preparation. After the above samples were washed three times, the supernatant was removed, and the precipitated fecal bacteria and sterile saline were dissolved and diluted in a 1:1 volume ratio and mixed to obtain the bacterial suspension. The bacterial suspension was then administered into the patient's intestinal cavity via a pre-placed digestive tract catheter (upper or lower). Each infusion delivered 120 mL of the bacterial solution. A single course of intestinal microbiota treatment consisted of one infusion per day for three consecutive days.

#### 2.3.3 Statistical analysis

In this study, SPSS 27.0 software was used for statistical analysis. The normality of all continuous data was assessed using the Shapiro–Wilk test. Since the majority of the biochemical marker data and clinical scores were found to be non-normally distributed (*p* < 0.05 in Shapiro–Wilk test) and/or due to the small sample sizes which limit the reliability of normality assumptions, we chose to use non-parametric statistics and present continuous data as median (interquartile range, IQR). The median and IQR are robust measures of central tendency and dispersion that are not influenced by extreme values or skewed distributions, providing a more accurate representation of our data. The 95% confidence intervals (95% CI) for median differences in all non-parametric tests were estimated using the Hodges–Lehmann method, and “estimated by Hodges–Lehmann method” was clearly indicated in result descriptions and tables to avoid only labeling “95% CI”.

Accordingly, the Mann–Whitney *U* test was used to assess differences in biochemical indexes between donors and AD patients. In addition to *p* values, effect sizes were calculated to quantify the magnitude of the observed differences or associations. For the Mann–Whitney *U* test, the effect size *r* was calculated as *r* = *Z*/√*N*, where *Z* is the test statistic and *N* is the total sample size. For the Wilcoxon signed-rank test, the effect size *r* was calculated as *r* = *Z*/√*N*, where *Z* is the test statistic and *N* is the number of paired observations. Effect sizes were interpreted according to Cohen's criteria: *r* ≈ 0.10 indicates a small effect, *r* ≈ 0.30 a medium effect, and *r* ≈ 0.50 a large effect.

The Wilcoxon signed-rank test was used to evaluate changes in intestinal barrier biomarkers and dermatitis scores before and after WMT treatment. Spearman's rank correlation test was used for correlation analyses, and the 95% CI of its correlation coefficient (*r_s_*) was calculated using the BCA Bootstrap method with 1,000 resamples to ensure the robustness of results. A *p* value less than 0.05 was considered statistically significant. To control for multiple comparisons, a stratified false discovery rate (FDR) correction was applied using the Benjamini-Hochberg procedure within three pre-defined analytical families: (1) the pathophysiology family (3 tests: group comparisons of the three intestinal barrier biomarkers between AD patients and healthy donors), (2) the intervention efficacy family (7 tests: pre- vs. post-WMT comparisons of the four clinical scores and the three biomarkers), and (3) the predictive exploration family (24 tests: all correlation analyses between baseline measures and treatment outcomes).The FDR was controlled independently within each family.

#### 2.3.4 Microbiota sequencing and analysis

Gut microbiota sequencing and analysis were completed by Majorbio Bio-Pharm Technology Co., Ltd. The specific procedures were as follows: First, genomic DNA was extracted, and the extraction quality was verified using 1% agarose gel electrophoresis. Subsequently, specific primers with barcodes were synthesized according to the designated sequencing region. Low-cycle amplification was performed using TransStart Fastpfu DNA Polymerase on an ABI GeneAmp 9,700 PCR instrument, with three technical replicates per sample followed by pooling of the products to minimize amplification bias. The amplification products underwent gel extraction recovery, fluorescence quantification, and were then mixed in proportional ratios for library construction before being sequenced on the Illumina Nextseq 2,000 platform. Following demultiplexing of the sequenced samples, paired-end reads were subjected to quality control, filtering, and assembly based on sequencing quality. The optimized data were subsequently processed using sequence denoising methods such as DADA2 to obtain Amplicon Sequence Variant (ASV) representative sequences and abundance information, followed by taxonomic annotation. To control for variations in sequencing depth across samples and enable comparative analysis of community diversity, all sample data were normalized to the same sequencing depth using rarefaction.

Analysis of the sequenced sample data was conducted as follows: Principal Coordinate Analysis (PCoA) based on the abund_jaccard distance matrix at the phylum and genus levels was performed to visualize inter-sample differences, and Analysis of Similarities (ANOSIM) with 999 permutations was used to assess the statistical significance of intergroup differences, with a *p*-value less than 0.05 considered statistically significant. The relative abundance of ASV at the phylum and genus levels was determined using the Naive Bayes algorithm, and bar charts and pie charts were generated to visualize the microbial community composition of the two groups (W0 vs. W1). Additionally, to identify bacterial genera showing significant differences before and after WMT treatment, the Wilcoxon signed-rank test was employed for species difference analysis at the genus level.

## 3 Results

### 3.1 Demographic characteristics 48 h prior to intestinal biochemical index testing

The donor group comprised 10 females (41.6%) and 14 males (58.4%). The AD patient group comprised 5 females (20.8%) and 19 males (79.2%). The mean age of donors was 21.16 ± 4.57 years, and the mean age of AD patients was 17.04 ± 7.96 years. The mean age of AD patients receiving WMT was 26.85 ± 17.18 years. None of the participants exhibited any relevant gastrointestinal symptoms, including diarrhea, nausea, vomiting, or abnormal bowel sounds, before the detection (Table 1).

### 3.2 Differences in biochemical indexes of intestinal barrier function between AD patients and healthy donors

Serum levels of D-lactate, endotoxin, and diamine oxidase were significantly elevated in AD patients compared to healthy donors (all *p* < 0.001), with large effect sizes (*r* = 0.643, 0.646, and 0.724, respectively). This confirms that AD patients have substantial intestinal barrier dysfunction (Table 2).

**Table 2:** Comparison of AD patients under 30 years old with donors (*n* = 47).

Biomarker	Group	Median (IQR)	Hodges–Lehmann Median Difference (95% CI)	*Z* value	Effect size (*r*)	*p* Value	FDR *q*-value
D-lactate (mg/L)	Donor	5.91 (3.33, 9.59)	−22.5 (−27.53, −8.09)	−4.724	0.689	2.30 × 10^−6^	2.30 × 10^−6^
	AD patient	31.44 (12.93, 35.90)					
Endotoxin (U/L)	Donor	5.99 (3.52, 11.77)	−12.83 (−16.04, −10.00)	−5.219	0.761	1.79 × 10^−7^	5.37 **× 10^−7^**
	AD patient	17.26 (13.18, 23.77)					
Diamine oxidase (U/L)	Donor	4.28 (1.10, 8.60)	−13.65 (−17.30, −10.07)	−4.852	0.707	1.22 × 10^−6^	1.83 × 10^−6^
	AD patient	18.17 (13.18, 24.87)					

(*n* = 47, Donor *n* = 23, AD patient *n* = 24) Group comparisons were performed using the Mann–Whitney *U* test. This family of tests (pathophysiology) comprised 3 hypotheses. All *P*-values remained statistically significant after False Discovery Rate (FDR) correction for these 3 tests (all *q* < 0.001).The normal reference ranges of D-lactate, endotoxin and diamine oxidase were <10 mg/L, <15 U/L and <20 U/L, respectively.

### 3.3 Comparison of biochemical indexes of intestinal barrier function in AD patients before and after one course of WMT treatment

**Figure 1:**
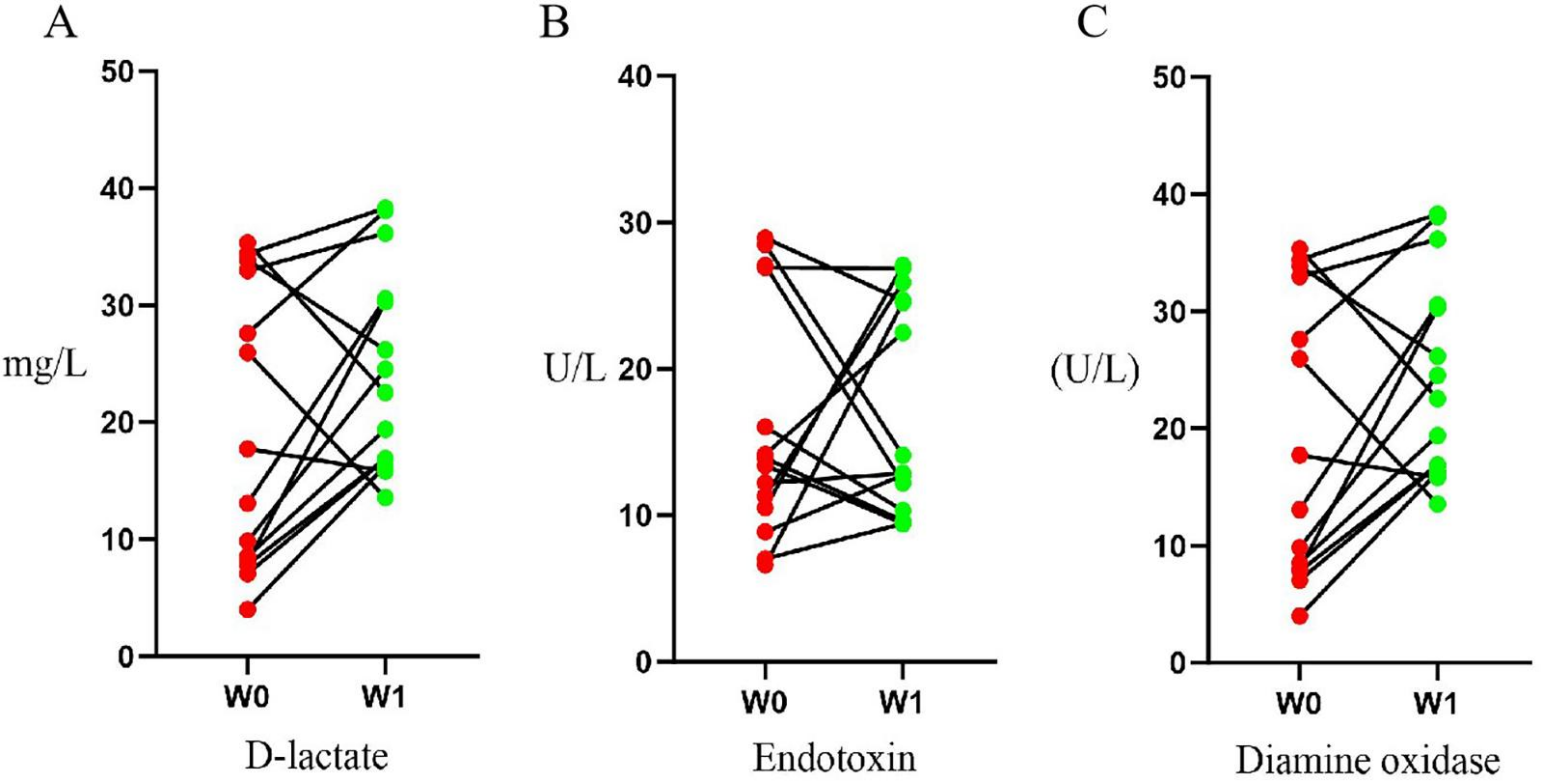
Serum levels of intestinal barrier biomarkers in patients with atopic dermatitis (AD) before and after washed microbiota transplantation (WMT). Paired measurements were taken at baseline (W0) and 4 weeks after WMT (W1). Individual data points are shown with lines connecting paired measurements for each patient. The biomarkers assessed were **(A)** D-lactate, **(B)** endotoxin, and **(C)** diamine oxidase (DAO)

No significant differences were observed in the biochemical indexes of intestinal barrier function in AD patients before and after a single course of WMT treatment (all *p* > 0.05; Table 3). The changes in diamine oxidase (*p* = 0.124, *r* = 0.411) and D-lactate (*p* = 0.096, *r* = 0.445) showed medium effect sizes, while the change in endotoxin was negligible (*p* = 0.826, *r* = 0.059). These results suggest that a single course of WMT did not produce significant short-term improvement in intestinal barrier function biomarkers (Table 3, Figure 1).

**Table 3:** Comparison of biochemical indexes of intestinal barrier function in AD patients before (W0) and four weeks after WMT treatment (W1).

Biomarker	Time point	Median(IQR)	Hodges–Lehmann Median Difference (95% CI)	*Z* value	Effect size (*r*)	*p* Value	FDR *q*-value
D-lactate (mg/L)	W0	15.42 (8.13, 27.19)	6.49 (−0.97, 12.26)	−1.663	0.445	0.096	0.168
	W1	20.98 (16.10, 30.50)					
Endotoxin (U/L)	W0	15.19 (14.70, 21.87)	0.85 (−4.97, 7.25)	−0.219	0.059	0.826	0.174
	W1	17.92 (16.48, 24.00)					
Diamine oxidase (U/L)	W0	17.47 (14.70, 22.66)	2.69 (−1.17, 7.37)	−1.538	0.411	0.124	0.826
	W1	17.25 (15.67, 23.27)					

*n* = 14. W0:before treatment; W1:4 weeks after treatment. The Wilcoxon signed-rank test was used for paired comparisons. The tests in this table are part of the pre-specified “intervention efficacy family”, which comprised 7 hypotheses in total (3 biomarkers 4 clinical scores). None of the changes reached statistical significance after FDR correction for these 3 tests (all q > 0.05).

### 3.4 The changes of rash and AD score in AD patients before and after the treatment of washed microbiota transplantation

**Figure 2:**
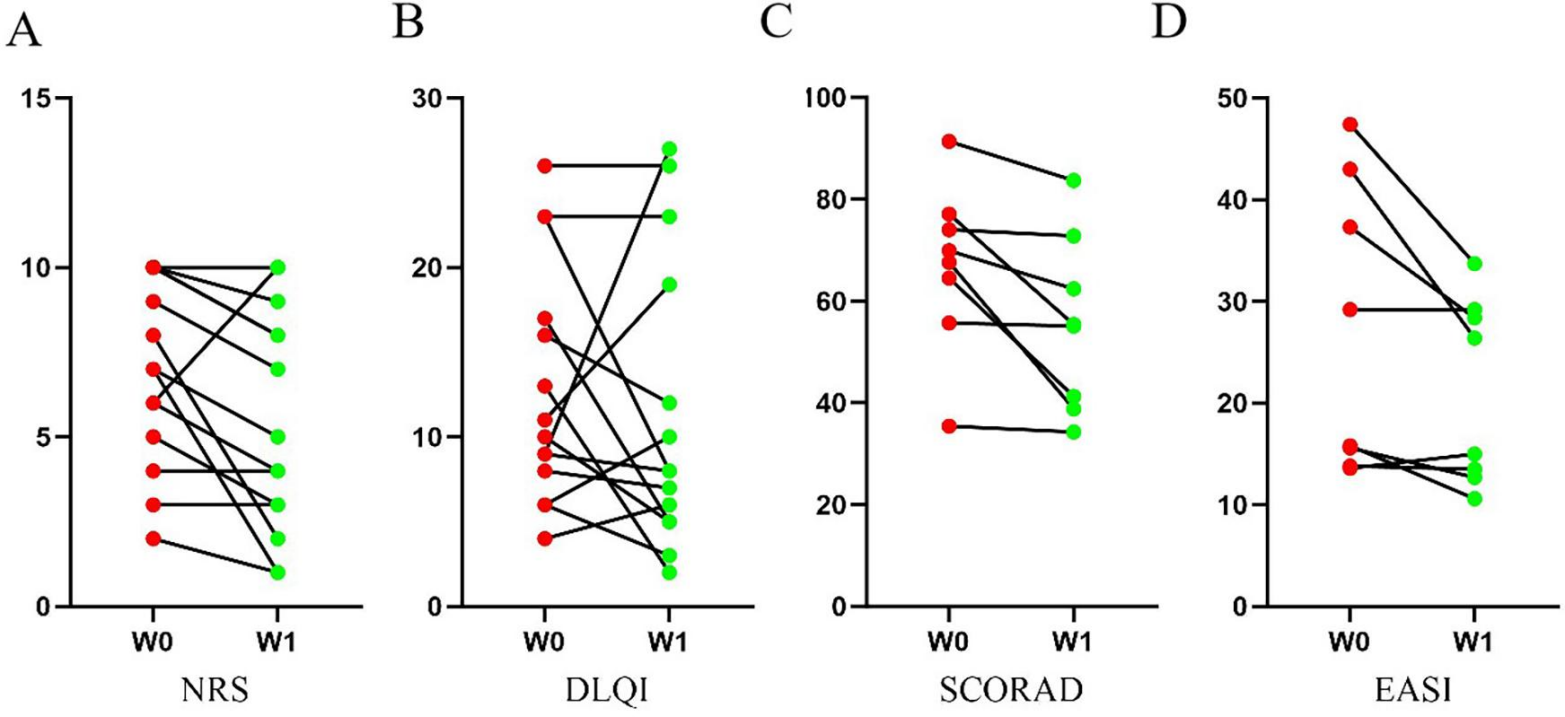
Clinical scores of patients with atopic dermatitis (AD) before and after washed microbiota transplantation (WMT).Paired measurements were taken at baseline (W0) and 4 weeks after WMT (W1). Individual data points are shown with lines connecting paired measurements for each patient. The clinical scores assessed were **(A)** NRS **(B)** (DLQI) **(C)** SCORAD **(D)** EASI.

**Figure 3:**
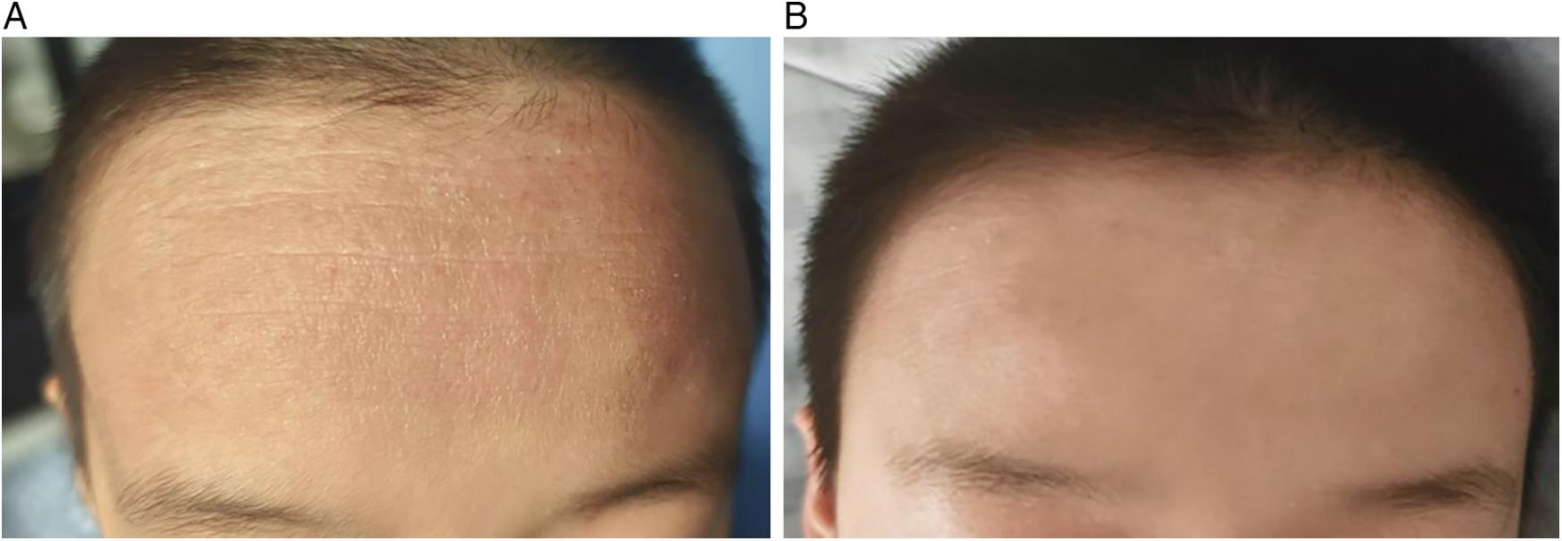
The changes of skin lesions in AD patients before and after WMT treatment. **(A)** Treatment of forehead rash. **(B)** Forehead rash after treatment.

Following WMT treatment, significant improvements were observed in SCORAD (*p* = 0.012, *r* = 0.891) and EASI (*p* = 0.018, *r* = 0.836) scores, with large effect sizes indicating robust improvements in clinical signs. The NRS score also decreased significantly (*p* = 0.030), albeit with a modest effect size (*r* = 0.581). In contrast, the improvement in the Dermatology Life Quality Index (DLQI) did not reach statistical significance (*p* = 0.410) and was associated with a small effect size (*r* = 0.220; Table 4, Figure 1). These results indicate that while AD severity, skin lesion area, and pruritus were significantly improved after four weeks of WMT, the short-term, patient-reported quality of life did not show a commensurate, statistically significant improvement (Table 4; Figures 2 and 3).

**Table 4:** The changes of AD score before (W0) and four weeks after WMT treatment (W1).

Score	Timepoint	Median (IQR)	Hodges–Lehmann Median Difference (95% CI)	*Z* value	Effect size (*r*)	*p* Value	FDR *q*-value
NRS (*n* = 14)	W0	7.50 (4.75, 9.75)	−1.50(−3.00, −0.50)	−2.173	0.581	0.030	0.105
	W1	4.50 (1.25, 7.75)					
DLQI (*n* = 14)	W0	12.00 (7.00, 21.25)	−5.55(−11.30, −0.150)	−0.824	0.220	0.410	0.478
	W1	7.00 (3.50, 17.25)					
SCORAD (*n* = 8)	W0	68.75 (57.90, 76.32)	−11.67(−22.45, −1.15)	−2.520	0.891	0.012	0.084
	W1	55.25 (39.42, 70.20)					
EASI (*n* = 8)	W0	22.50 (14.25, 41.57)	−1.50(−6.50, 3.00)	−2.028	0.717	0.043	0.100
	W1	19.95 (11.12, 29.00)					

W0: before treatment; W1: 4 weeks after treatment. SCORAD. The Wilcoxon signed-rank test was used for paired comparisons. The tests in this table are part of the pre-specified “intervention efficacy family”, which comprised 7 hypotheses in total (3 biomarkers in Tables 3 and 4 clinical scores in this table).

### 3.5 Correlation analysis between intestinal barrier function and dermatitis score in AD patients

No significant correlations were found between pre-WMT levels of D-lactate, endotoxin, or diamine oxidase and pre-treatment SCORAD, EASI, NRS, or DLQI scores in AD patients (all *p* > 0.05; Table 5). It is important to note that these analyses, part of an exploratory family of tests, did not survive correction for multiple comparisons (FDR-adjusted *p* > 0.05 for all).

**Table 5:** Correlation analysis between intestinal barrier function before WMT and AD score.

Biomarker before WMT	AD Score before WMT	Spearman's *ρ* (95% CI)	*p* Value	FDR *q*-value
D-lactate (mg/L)	NRS (*n* = 14)	−0.118 (0.468, 0.615)	0.688	1.000
	DLQI (*n* = 14	−0.042 (−0.752, 0.506)	0.887	0.967
	SCORAD (*n* = 8)	0.167 (−0.644, 0.778)	0.693	1.000
	EASI (*n* = 8)	0.071 (−0.797, 0.848)	0.867	1.000
Endotoxin (U/L)	NRS (*n* = 14)	−0.254 (0.751, 0.357)	0.381	1.000
	DLQI (*n* = 14)	−0.223 (−0.808, 0.408)	0.444	1.000
	SCORAD (*n* = 8)	−0.2149 (−0.776, 0.468)	0.610	1.000
	EASI (*n* = 8)	−0.429 (−0.975, 0.640)	0.289	1.000
Diamine oxidase (U/L)	NRS (*n* = 14)	0.145 (−0.468, 0.615)	0.621	1.000
	DLQI (*n* = 14	0.053 (−0.560, 0.629)	0.857	1.000
	SCORAD (*n* = 8)	0.048 (−0.772, 0.747)	0.911	0.950
	EASI (*n* = 8)	0.071 (−1.000, 0.722)	0.867	0.990

Note. W0: before treatment; W1: 4 weeks after treatment. Note. The tests in this table are part of the pre-specified “predictive exploration family”, which comprised 24 hypotheses in total.

### 3.6 Analysis of the correlation between intestinal barrier function and the efficacy of WMT in the treatment of AD

In an exploratory analysis of potential predictive biomarkers, we found that pre-treatment D-lactate levels showed a strong, uncorrected correlation with the changes in NRS and SCORAD scores. Specifically, a significant negative correlation was observed between pre-treatment D-lactate level and the NRS score change (*r* = −0.650, *p* = 0.012). Similarly, a significant negative correlation was found with the SCORAD score change (*r* = −0.738, *p* = 0.037). However, after applying a stratified False Discovery Rate (FDR) correction to the entire family of 24 exploratory correlation tests, these associations were no longer statistically significant. Therefore, these findings, while internally consistent and hypothesis-generating, cannot be considered definitive and require validation in a larger, independent cohort. No other significant correlations, either uncorrected or after FDR correction, were observed between baseline biomarkers and clinical score changes (Table 6).

**Table 6:** Correlation analysis between intestinal barrier function before WMT and AD score difference after WMT treatment.

Biomarker before WMT	Score difference after WMT	Spearman's *ρ* (95% CI)	*p* Value	FDR *q*-value
D-lactate (mg/L)	NRS (*n* = 14)	−0.650	0.012	0.288
	DLQI (*n* = 14	−0.286	0.321	1.000
	SCORAD (*n* = 8)	−0.738	0.037	0.444
	EASI (*n* = 8)	0.452	0.260	1.000
Endotoxin (U/L)	NRS (*n* = 14)	−0.069	0.814	1.000
	DLQI (*n* = 14	0.236	0.417	1.000
	SCORAD (*n* = 8)	−0.190	0.651	1.000
	EASI (*n* = 8)	0.500	0.207	1.000
Diamine oxidase (U/L)	NRS (*n* = 14)	−0.184 (−0.853, 0.435	0.528	1.000
	DLQI (*n* = 14	0.026 (−0.726, 0.720)	0.929	0.929
	SCORAD (*n* = 8)	−0.310 (−0.921, 0.782)	0.456	1.000
	EASI (*n* = 8)	0.095 (−1.000 ,0.926)	0.823	1.000

The tests in this table are part of the pre-specified “predictive exploration family”, which comprised 24 hypotheses in total.Score difference = 4 weeks after treatment (W1)-before treatment (W0).

### 3.7 Analysis results of intestinal microbiota

**Figure 4:**
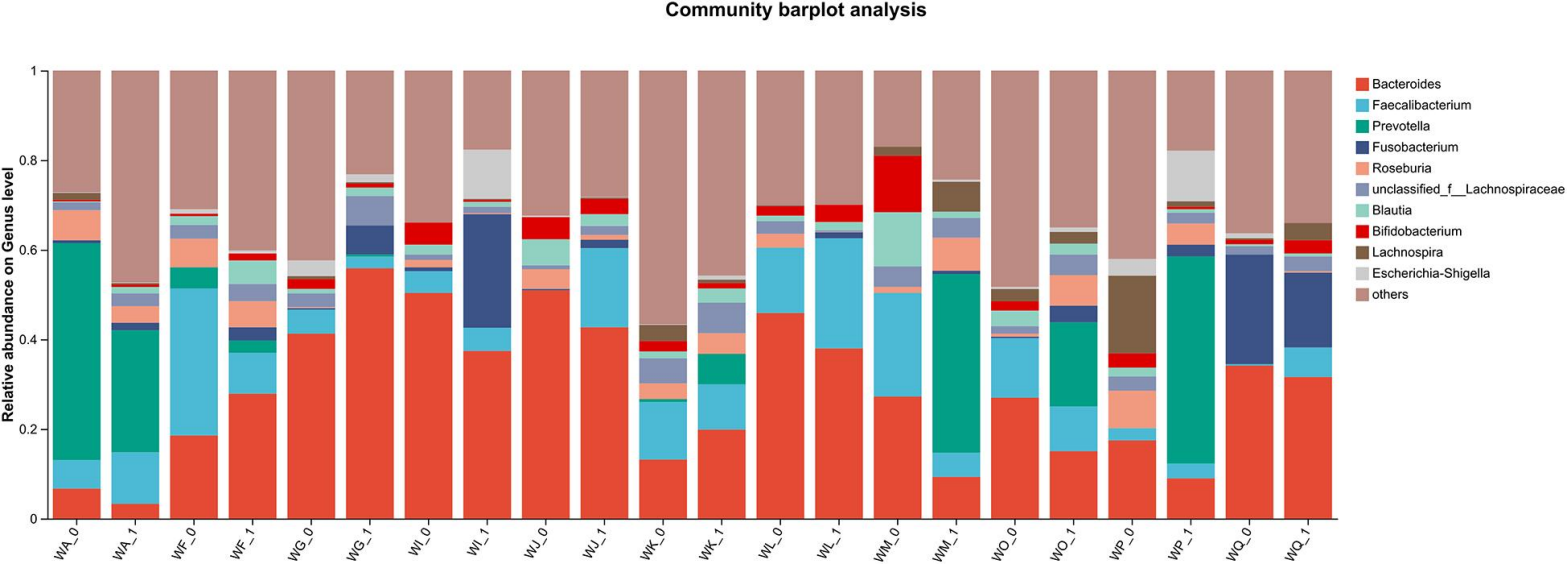
Bar chart showing bacterial composition at the genus level in AD patients before (W0) and after (W1) WMT treatment.

**Figure 5:**
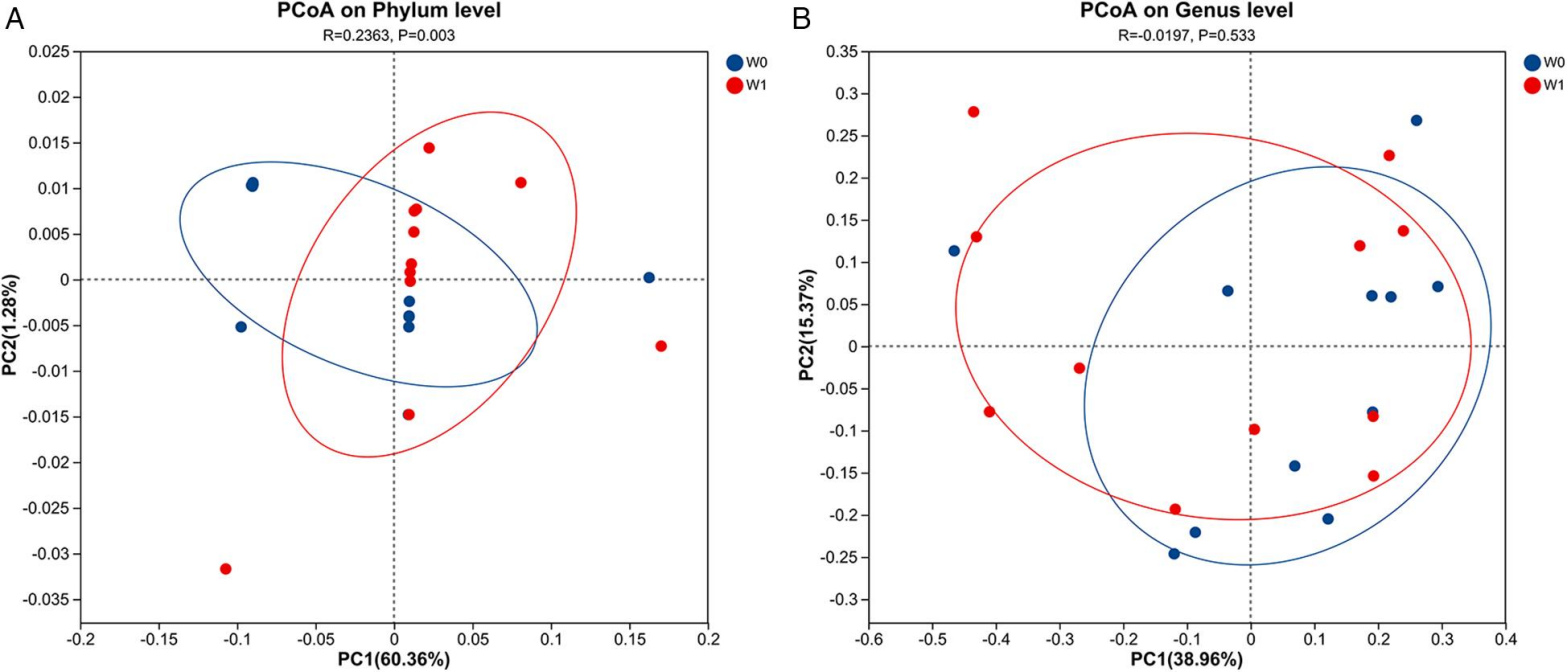
PCoA at the phylum **(A)** and genus **(B)** level. The percentage in parentheses on each axis label (PC1/PC2) represents the proportion of total variation explained by that principal coordinate. A higher percentage indicates that the axis is more important in distinguishing sample differences. The “R” value is derived from PERMANOVA testing, reflecting the extent to which predefined groups explain the observed community variation. A “P” value less than 0.05 indicates statistically significant differences between groups.

**Figure 6:**
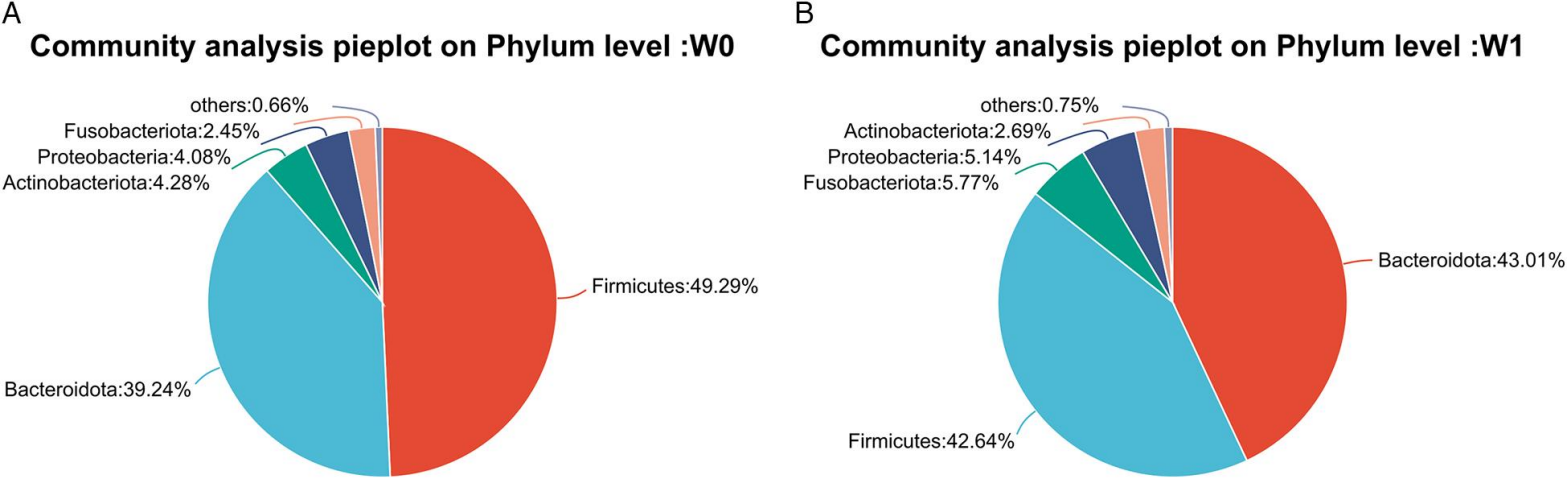
Pie charts depicting microbiota composition at the phylum level in AD patients **(A)** before (W0) and **(B)** after (W1) WMT treatment.

**Figure 7:**
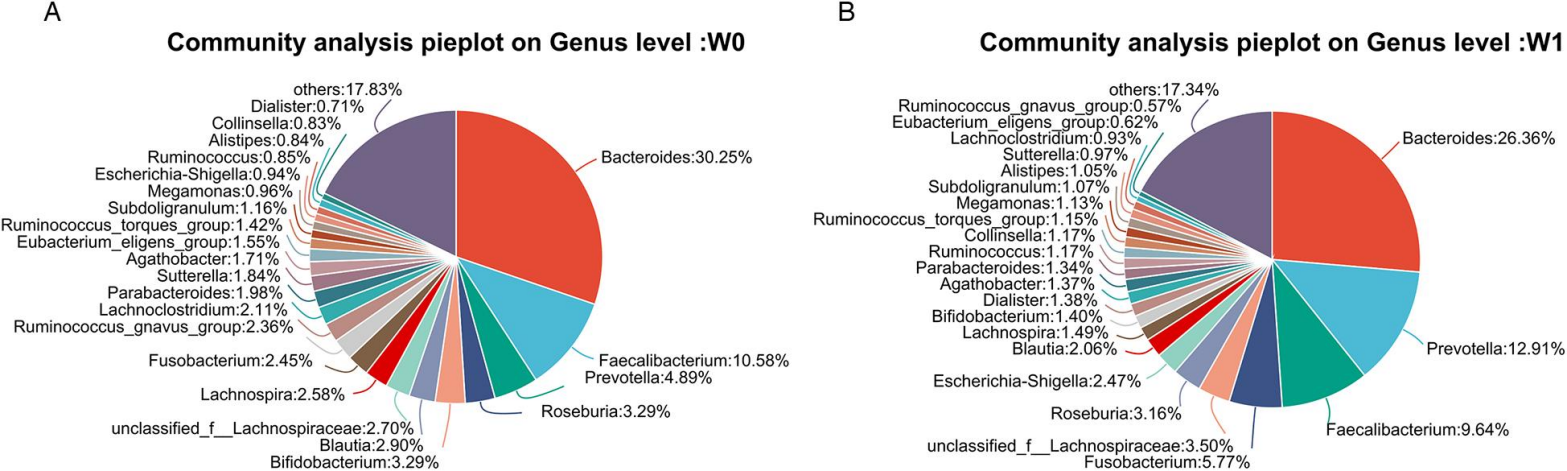
Pie charts depicting microbiota composition at the genus level in AD patients **(A)** before (W0) and **(B)** after (W1) WMT treatment.

Fecal samples collected before (W0) and after four weeks of WMT treatment (W1) from 11 AD patients underwent bacterial sequencing and were analyzed at the phylum and genus levels. As it was difficult to discern a unified change trend for individual bacterial species (Figure 4). PCoA at the phylum level indicates a significant difference in microbial community structure before and after WMT treatment (*R* = 0.2363, *p* = 0.003), while the difference at the genus level is not significant (*R* = −0.0197, *p* = 0.533) (Figure 5).Group comparisons (W0 vs. W1) were used to describe overall microbiota changes. At the phylum level (Figure 6), the relative abundances of *Bacteroidota*, *Proteobacteria*, and *Fusobacteriota* showed an increasing trend, rising from baseline values of 39.24%, 4.08%, and 2.45% to 43.01%, 5.14%, and 5.77%, respectively. In contrast, the abundances of *Firmicutes* and *Actinobacteria* decreased significantly, declining from 49.29% and 4.28% to 42.64% and 2.69%, respectively. At the genus level (Figure 7), several key alterations were observed. Most strikingly, the abundance of *Prevotella* increased markedly from 4.89% to 12.91%. *Fusobacterium* also increased from 2.45% to 5.77%, and *Escherichia-Shigella* from 0.94% to 2.47%. In contrast, the core genus *Bacteroides* decreased from 30.25% to 26.36%. Several other genera exhibited decreased abundances: *Ruminococcus_gnavus_group* declined from 2.36% to 0.57%, *Lachnospira* from 2.58% to 1.49%, *Blautia* from 2.90% to 2.06%, and *Bifidobacterium* from 3.29% to 1.40%. Notably, the beneficial genera *Faecalibacterium* and *Roseburia*, known as short-chain fatty acid producers, remained relatively stable, with *Faecalibacterium* showing a slight decrease from 10.58% to 9.64% and *Roseburia* from 3.29% to 3.16%.

**Figure 8:**
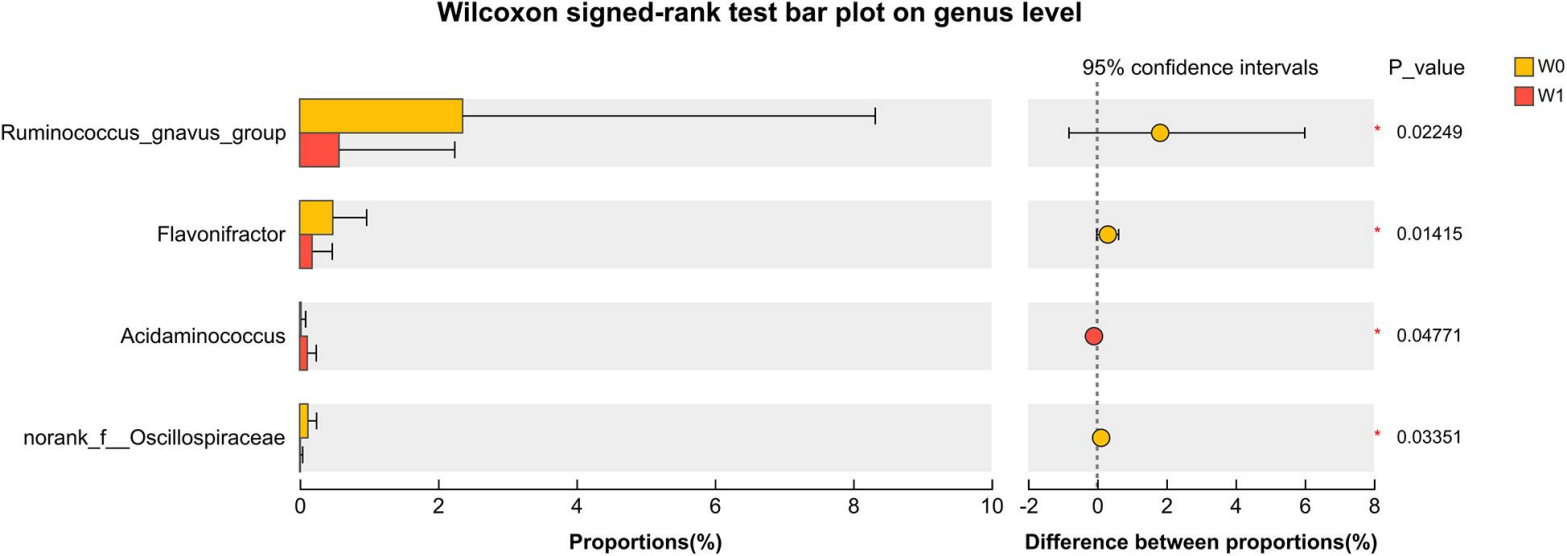
Genus-level differential species analysis.the left panel displays the relative abundance of bacterial genera at baseline (W0) and four weeks after WMT treatment (W1), while the right panel shows the 95% confidence intervals for the differences in abundance. Statistical analysis was performed using the Wilcoxon signed-rank test, with a *p*-value less than 0.05 considered statistically significant.

Differential abundance analysis at the genus level demonstrated significant alterations in the gut microbiota after WMT, characterized by an increase in *Acidaminococcus* and decreases in *Ruminococcus_gnavus_group*, *Flavonifractor*, and *Norank_f_Oscillospiraceae* (Figure 8).

## 4 Discussion

Our study demonstrates that patients with AD exhibit significant intestinal barrier dysfunction and that WMT can effectively improve its clinical symptoms. The most novel finding is the strong correlation between pre-treatment intestinal permeability, reflected by serum D-lactate levels, and the clinical efficacy of WMT, suggesting its potential as a predictive biomarker. However, it is critical to emphasize that this correlation, while strong in uncorrected analysis, did not survive strict False Discovery Rate (FDR) correction for multiple comparisons in our exploratory analysis. Therefore, it must be strictly interpreted as a preliminary, hypothesis-generating signal that requires validation in larger, dedicated cohorts.

The baseline barrier dysfunction in these AD patients likely stems from a known pathophysiological cycle: characteristic Th2 immune deviation (e.g., IL-4/IL-13 overexpression) can directly inhibit the synthesis of tight junction proteins like Claudin-1 (24, 25), while other inflammatory mediators may exacerbate epithelial damage (26). We hypothesize that the early clinical benefits of WMT are mediated through rapid immunomodulation rather than immediate structural barrier repair. The pre-existing state of increased intestinal permeability might facilitate the translocation of beneficial microbial metabolites derived from the newly transplanted microbiota into systemic circulation, thereby directly influencing systemic inflammation and skin immunity. This could mechanistically explain why patients with higher baseline D-lactate levels experienced greater symptom improvement in our uncorrected analysis. This concept is supported by studies showing that SCFAs, such as butyrate, can suppress pro-inflammatory pathways and inhibit NF-*κ*B, even in the absence of barrier restoration (27, 28).

Concurrent with clinical improvement, our genus-level differential abundance analysis revealed a functionally coherent restructuring of the gut ecosystem, moving towards a profile with enhanced barrier-protective and anti-inflammatory potential. A pivotal finding was the significant decrease in *Ruminococcus_gnavus_group*, a genus recognized for its ability to degrade intestinal mucin, a process that can compromise gut barrier integrity and activate immune responses (29). Its abundance is notably elevated in infants with allergic diseases and correlates with inflammation precursors (30).The reduction of this pro-inflammatory, barrier-disrupting genus post-WMT likely represents a crucial step in mitigating the gut-skin axis of inflammation in AD.

This suppression of a detrimental taxon was strategically accompanied by a beneficial enrichment. We observed a marked increase in *Acidaminococcus*, which can degrade proteins and amino acids to generate SCFAs, notably propionate and butyrate. These metabolites can inhibit the NF-*κ*B pathway, reduce pro-inflammatory cytokines such as IL-6, and promote the secretion of anti-inflammatory IL-10 (31). The co-occurrence of these changes—the suppression of a barrier-disrupting, pro-inflammatory genus (R. gnavus) alongside the enrichment of a SCFA-producing genus (Acidaminococcus)—suggests that WMT orchestrates a targeted restoration of gut ecological function.

The treatment also induced a significant reduction in *Flavonifractor*, a genus whose role in AD is complex and not yet fully understood (32). Its decrease in our cohort may indicate a beneficial shift away from a dysbiotic state. Similarly, the decline in *Norank_f_Oscillospiraceae*, a common yet poorly characterized member of the *Firmicutes* phylum, adds another layer to this ecological restructuring. While its functional contribution remains unclear, its reduction alongside other pro-inflammatory *Firmicutes* points to a broad, potentially beneficial reorganization within this phylum. The net functional outcome of this new ecosystem is likely more critical than the change in any single genus, potentially creating a microbial community with an enhanced capacity to produce anti-inflammatory metabolites and a reduced capacity to provoke immune activation.

The interpretations of our study must be tempered by its acknowledged limitations. The single-arm, non-blinded design and the modest sample size, particularly in the WMT cohort, preclude definitive causal inferences and limit the generalizability of our findings. The promising correlation between D-lactate and clinical outcomes, while internally consistent, requires independent validation in a larger cohort and did not survive multiple comparison corrections. The 4-week follow-up was sufficient to capture early clinical response but was likely too short to observe the full timeline of microbial and barrier stabilization. Finally, the absence of direct immune or metabolomic measurements means our proposed immunomodulatory mechanism remains speculative and is presented as a testable hypothesis for future research.

In summary, this study confirms significant intestinal barrier dysfunction in AD and demonstrates the potential clinical efficacy of WMT. The strong, uncorrected correlations suggest that pre-treatment D-lactate level warrants further investigation as a candidate biomarker for predicting WMT response. Critically, the dissociation between significant clinical improvement and the absence of short-term barrier restoration reframes our understanding of WMT's mechanism. Our findings suggest that the early efficacy of WMT is likely driven by rapid immunomodulation, mediated by a restructured gut microbiota that suppresses pro-inflammatory taxa and enhances beneficial metabolic potential, rather than by immediate structural repair.

## 5 Conclusion

In conclusion, this study confirms intestinal barrier dysfunction in atopic dermatitis (AD) and provides preliminary evidence for the efficacy of Washed Microbiota Transplantation (WMT). The strong, uncorrected correlation between pre-treatment D-lactate levels and clinical improvement identifies it as a candidate predictive biomarker, warranting future validation. The dissociation between rapid symptom relief and lack of immediate barrier repair suggests that early WMT efficacy is driven by immunomodulation, potentially facilitated by the pre-existing permeable gut environment and a restructured gut microbiota. These findings advance our understanding of microbiota-targeted therapies for AD.

## Data Availability

The datasets presented in this study can be found in online repositories. The names of the repository/repositories and accession number(s) can be found below: https://www.ncbi.nlm.nih.gov/, PRJNA1364770.
